# ANGPTL3 Is Involved in the Post-prandial Response in Triglyceride-Rich Lipoproteins and HDL Components in Patients With Coronary Artery Disease

**DOI:** 10.3389/fcvm.2022.913363

**Published:** 2022-06-29

**Authors:** Xin Guo, Zhijie Huang, Jin Chen, Jiarui Hu, Die Hu, Daoquan Peng, Bilian Yu

**Affiliations:** ^1^Department of Cardiovascular Medicine, Research Institute of Blood Lipid and Atherosclerosis, The Second Xiangya Hospital, Central South University, Changsha, China; ^2^Department of Spine Surgery, The Second Xiangya Hospital, Central South University, Changsha, China

**Keywords:** angiopoietin-like protein 3, coronary artery disease, triglyceride-rich lipoproteins, high-density lipoprotein, post-prandial state

## Abstract

It is well-established that there exists an inverse relationship between high-density lipoprotein (HDL) cholesterol and triglyceride (TG) levels in the plasma. However, information is lacking on the impact of post-prandial triglyceride-rich lipoproteins (TRLs) on the structure of HDL subclasses in patients with coronary artery disease (CAD). In this study, the data of 49 patients with CAD were analyzed to evaluate dynamic alterations in post-prandial lipid profiles using nuclear magnetic resonance-based methods. An enzyme-linked immunosorbent assay was used to quantify the serum angiopoietin-like protein 3 (ANGPTL3). After glucose supplementation, the expression of hepatic ANGPTL3 was evaluated both *in vitro* and *in vivo*. Compared to fasting levels, the post-prandial serum TG level of all participants was considerably increased. Although post-prandial total cholesterol in HDL (HDL-C) remained unchanged, free cholesterol in HDL particles (HDL-FC) was significantly reduced after a meal. Furthermore, the post-prandial decrease in the HDL-FC level corresponded to the increase in remnant cholesterol (RC), indicating the possible exchange of free cholesterol between HDL and TRLs after a meal. Moreover, CAD patients with exaggerated TG response to diet, defined as TG increase >30%, tend to have a greater post-prandial increase of RC and decrease of HDL-FC compared to those with TG increase ≤30%. Mechanistically, the fasting and post-prandial serum ANGPTL3 levels were significantly lower in those with TG increase ≤30% than those with TG increase >30%, suggesting that ANGPTL3, the key lipolysis regulator, may be responsible for the different post-prandial responses of TG, RC, and HDL-FC.

## Introduction

Increasing evidence has indicated that triglyceride-rich lipoproteins (TRLs), such as chylomicrons, very-low-density lipoprotein, and intermediate-density lipoprotein, may considerably contribute to residual cardiovascular risk in individuals with coronary artery disease (CAD), despite statin use (1–3). Post-prandial dyslipidemia manifests as markedly elevated TRL and remnant cholesterol (RC) levels, and thus increases the likelihood of atherosclerotic cardiovascular disease (4). This well-established post-prandial phenomenon, frequently displayed as a strong negative connection between the plasma concentrations of HDL-C and triglycerides, indicates a possible association between HDL and TRL metabolism. Conversely, the essential metabolic connection between them remains poorly known.

Many studies have found that a low HDL-C level is strongly linked to greater CAD risk in humans. However, exceptionally high HDL-C levels are equally associated with increased CAD risk, leading to a U-shaped relationship between HDL-C and CAD (5). Thus, it is necessary to ascertain the modification processes of HDL components rather than HDL-C levels. HDL components, derived from a variety of sources, undergo significant changes in the post-prandial periods. Following a meal, the liver and small intestine produce and secrete apolipoprotein A1 (Apo-A1) and nascent HDL. Intravascular HDL and TRLs interact through various metabolic pathways, most of which contain hetero-exchange of core lipids by cholesteryl ester transfer protein (CETP), resulting in the formation of triglyceride-enriched HDL and cholesteryl ester-enriched TRLs (6). In addition, lipoprotein lipase (LPL)-mediated lipolytic catabolism of TRLs triggers the transfer of excessive surface components, such as free cholesterol (FC), phospholipid (PL), and apolipoproteins (such as Apo-A1), to HDL, thus enriching the HDL mass (7). Indeed, in this commonly neglected process, high amounts of free cholesterol are delivered to HDL, which is the principal source of circulating HDL-C (8). Subsequently, remnant-derived free cholesterol in HDL is transferred to the liver for excretion, in a process known as reverse RC transport (RRT) (9). Lipolysis of LPL-mediated TRLs is the rate-limiting process during RRT. It has been reported that fluctuations in LPL activity could explain a significant proportion of the variability in HDL-C levels (10). Therefore, the lipolytic activity in the post-prandial state and its impact on the HDL lipid content, especially HDL-C level, are worth discussing. However, in addition to LPL, numerous other factors contribute to the lipolysis of TRLs. For example, glycosylphosphatidylinositol-anchored high-density lipoprotein-binding protein 1, a GPI-anchored protein found in capillary endothelial cells, delivers LPL to its site of action in the capillary lumen, which is essential for the lipolysis of TRLs (11). However, detecting the activity of a single enzyme cannot completely reflect the action of the lipolytic processing of TRLs in the post-prandial state. Thus, it would be interesting to explore a simple biochemical marker reflecting the lipolytic activity of TRLs.

Unlike angiopoietin-like protein 8 (ANGPTL8), which mainly inhibits LPL and affects TG metabolism, ANGPTL3 could inhibit LPL and endothelial lipase (EL) and plays an important role in both TG and HDL metabolism (12). ANGPTL3 inhibition of LPL requires complex formation with ANGPTL8, which is not required for its inhibition of EL (13). Individuals with ANGPTL3 loss-of-function alleles have low plasma TG, LDL-C, and HDL-C levels and decreased risk of CAD (14). Some studies have shown that ANGPTL3 can impact HDL-C levels by inhibiting phospholipase EL (15, 16). Zhao et al. (17) found that ANGPTL3 was related to HDL components and function in female participants without diabetes. Conversely, the impact of ANGPTL3 on plasma HDL metabolism has not been adequately explored. Most studies have demonstrated that nuclear magnetic resonance (NMR) metabolomics can be used to effectively characterize lipoprotein composition (18, 19). Previous studies have also revealed that the NMR-based method is preferable to the enzymatic method when evaluating cholesterol in LDL and TRLs in the post-prandial state (20, 21). The lipids and proteins in HDL vary in response to disease development, lipid-modulating therapy, and metabolic changes. In this study, an NMR-based method was used to characterize the post-prandial effects of ANGPTL3 on HDL composition in patients with CAD using statins. Considering the RRT hypothesis (9), it would be interesting to explore whether ANGPTL3, a lipolysis inhibitor, could regulate HDL metabolism by accelerating the lipolysis of TRLs. Moreover, dyslipidemia after a meal is similar to the lipid profiles observed in type-2 diabetes mellitus. Whether glucose metabolism plays a role in the ANGPTL3-associated link between HDL and TRL metabolism during the post-prandial period remains elusive.

## Methods

### Participants

Forty-nine participants were enrolled from the Second Xiangya Hospital of Central South University from August to November 2019. The detailed criteria for the diagnosis of CAD and the exclusion criteria for participants have been previously described (20).

### Blood Sample Collection

As described in a previous study (22), all participants were permitted to have a standard Chinese breakfast to mimic physiological states. A typical Chinese breakfast comprises steamed buns, milk, eggs, noodles, and soups with a small amount of oil. The time of intake, quantity consumed in portion sizes, and preparation form of all foods and beverages were individually documented. The energy value generally ranged from 500 to 600 kcal, with 8–10% fat, 45–55% carbohydrate, and 15–20% protein content. Peripheral venous blood was collected in the fasting state (after overnight fasting for at least 10 h) and at the 2 and 4 h post-prandial states.

### Clinical and Biochemical Measurement

Researchers recorded all participants' baseline clinical information (sex, age, statin use, smoking, drinking, diabetes, and hypertension). Weight and height data were assessed after overnight fasting (for at least 10 h). Briefly, blood samples were diluted 1:20, and the serum ANGPTL3 levels of each sample were quantified using an ELISA kit (Abcam, ab254510). The serum insulin levels were quantified using an ELISA kit (Proteintech, KE00045). The human serum free fatty acid (FFA) and mouse lipid profiles were analyzed through standard laboratory procedures using Roche automated clinical chemistry analyzers. The total plasma lipid profiles (TG, TC, LDL-C, TRL, and HDL lipid content) were measured at ProteinT Biotechnology Co., Ltd. (Tianjin, China) using a Bruker 600 MHz Avance III NMR spectrometer as previously described (20). Standardization of the spectra to the same quantitative scale was performed using the Bruker's QuantRef manager within TopSpin according to the PULCON approach (23), which revealed exceptional reproducibility, with an inter-coefficient variation of 1.39–2.62% (23). As previously described (20), the equation for estimating RC *via* NMR is: very-low-density lipoprotein (VLDL) 3-C + VLDL5-C + VLDL4-C + intermediate-density lipoprotein (IDL)-C. The equation used to assess cholesterol in TRL (TRL-C) with NMR was: TRL-C = TC-(HDL-C + LDL-C).

### Animals

Eight-week-old male C57BL/6J mice were acquired from SJA Laboratory Animal Co., Ltd. (Hunan, China) and maintained at 22 ± 1°C, under a 12-h light/ dark cycle. Before the tests, all mice were allowed free access to regular laboratory diet and water. All mice were equally separated into fasted and re-fed groups for the fasting and refeeding experiments. Before the study commenced, all groups were in a 16 h fasted state. The re-fed group was fed a high-carbohydrate, low-fat diet (Rodent Diet with 10 kcal% fat; Research Diets, D12450J) for 3 h. Blood and liver samples of mice were then harvested and stored for subsequent examination.

### Cell Culture

HepG2 cells were obtained from ATCC (ATCC, HB-8065) and cultured in DMEM supplemented with 10% FBS and 1% penicillin/streptomycin (Gibco, #15140122). For the glucose supplementation experiments, HepG2 cells were incubated in insulin-free DMEM without glucose for 12 h and then changed to low-glucose (5 mM) or high-glucose (25 mM) DMEM for times indicated in the figure legend.

### Total RNA Isolation, cDNA Synthesis, and Quantitative Real-Time PCR Analyses

Total RNA was extracted from tissues using TRIzol (Invitrogen, #15596026), and GeneJET RNA purification kits (Thermo Fisher Scientific, K0731) were used to extract total RNA from the cells. To synthesize cDNA, a RevertAid First Strand cDNA Synthesis Kit (Thermo Fisher Scientific, K1622) was utilized. The quantitative real-time PCR studies were carried out on a Bio-Rad CFX Connect Real-time System (Bio-Rad) with a 15 μL reaction system. The primer sequences are presented in Supplementary Table 1. The 2-ΔΔCT method was carried out to calculate the relative quantification of gene expression normalized to that of β-actin.

### Transfection of siRNA

For gene silencing assays, HepG2 cells were transfected with ChREBP-specific siRNA during incubation at a final siRNA dose of 10 nM using Lipofectamine RNAiMAX transfection reagent (Invitrogen, #13778030). Simultaneously, a scrambled siRNA was transfected as a negative control.

### Statistical Analysis

Normally distributed continuous data are shown as the mean ± SEM. Repeated-measures analysis of variance or two-way ANOVA with multiple comparisons was performed to analyze differences among three groups or more. Comparisons between two groups were performed by unpaired two-tailed Student's *t*-test. Non-normally distributed data are described using medians and interquartile ranges. Logarithmic transformation was performed when necessary. Further, comparison of data with a non-normal distribution was performed with generalized estimating equations. Spearman's correlation and multiple linear regression (MLR) were used to determine the association between lipid and glucose levels and ANGPTL3 levels. Before MLR analysis, serum ANGPTL3, TG, TC, FFA, and glucose levels were log-transformed to be normally distributed. Because they were not normally distributed. Statistical significance was set at *P* < 0.05.

## Results

### Participant Characteristics

To investigate the metabolic link between HDL and TRLs in patients with CAD in the post-prandial state, 49 participants with CAD were enrolled to evaluate the fasting, 2-h-post-prandial, and 4-h-post-prandial HDL and TRL components using an NMR-based method. The participants' median age was 59.44 ± 1.42 years, and 90% were male. Hypertension and type-2 diabetes mellitus were present in 57.1 and 38.8% of participants, respectively. Five (26.3%) diabetic participants didn't receive hypoglycemic treatment. Statins were used by 100% of the participants. Despite statin use, 40.8% of participants exhibited an abnormal TG response to meals, defined as a post-prandial increase in serum TG levels >30% according to our previously published data (22). Thus, according to the percentage of post-prandial serum TG rise, the 49 participants were separated into two groups: ΔTG ≤ 30% and ΔTG > 30%. The basic clinical characteristics of the participants are summarized in Table 1. Baseline demographics, fasting glucose, insulin, and lipid profiles did not differ significantly between the ΔTG ≤ 30% and ΔTG > 30% groups. Notably, the levels of FFA, the products of lipolysis, were significantly higher in the ΔTG ≤ 30% group than in the ΔTG > 30% group, suggesting a higher lipolysis ability in the ΔTG ≤30% group [0.33 (0.24–0.47) vs. 0.21 (0.11–0.35) mmol/L, *p* = 0.025].

**Table 1:** Baseline characteristics and lipoprotein summaries.

**Variable**	**All (*n* = 49)**	**Percentage of post-prandial plasma TG elevation**		***P*** **value**
		**ΔTG ≤ 30% (*n* = 29)**	**ΔTG > 30% (*n* = 20)**	
Age (years)	59.45 ± 1.42	58.55 ± 1.87	60.75 ± 2.18	0.392^b^
Sex, male	44 (89.8)	27 (93.1)	17 (85.0)	0.659^a^
Hypertension	28 (57.1)	17 (58.6)	11 (55.0)	0.801^a^
Diabetes	19 (38.8)	14 (48.3)	5 (25.0)	0.100^a^
Smoking	26 (53.1)	17 (58.6)	9 (45.0)	0.348^a^
Drinking	11 (22.4)	4 (13.8)	7 (35.0)	0.161^a^
BMI (kg/m^2^)	24.64 ± 0.54	23.95 ± 0.59	25.55 ± 0.97	0.147^b^
Hypoglycemic drug usage	14 (28.6)	11 (37.9)	3 (15.0)	0.827^a^
Metformin	8 (16.3)	7 (24.1)	1 (5.0)	0.338^a^
α-GI	8 (16.3)	6 (20.7)	2 (10.0)	1.000^a^
SGLT2	6 (12.2)	6 (20.7)	0	0.128^a^
Insulin therapy	4 (8.2)	4 (13.8)	0	0.530^a^
**Baseline lipids**				
TC (mg/dL)	152.19 (129.53–180.09)	152.19 (131.13–187.51)	153.23 (126.61–172.08)	0.579^b^
LDL-C (mg/dL)	71.88 ± 4.59	70.23 ± 5.94	74.26 ± 7.37	0.670^b^
HDL-C (mg/dL)	43.39 ± 1.13	42.18 ± 1.24	45.13 ± 2.09	0.204^b^
TG (mg/dL)	141.68 (80.94–199.37)	144.40 (105.24–239.75)	97.62 (78.47–189.86)	0.255^c^
RC (mg/dL)	22.82 ± 2.09	26.29 ± 3.04	17.78 ± 2.22	0.078^c^
FFA (mmol/L)	0.27 (0.19–0.43)	0.33 (0.24–0.47)	0.21 (0.11–0.35)	0.025^*^^c^
Glucose (mmol/L)	4.60 (4.10–6.30)	5.00 (4.15–6.75)	4.35 (4.10–5.18)	0.089^c^
HbA1c (%)	6.10 (5.70–7.30)	6.00 (2.50–7.30)	6.00 (5.50–6.20)	0.108^c^
Insulin (pmol/L)	134.98 (92.60–246.32)	131.33 (91.59–249.28)	154.35 (94.15–248.85)	0.620^c^

*Data are shown as median (low quartile-upper quartile), n (%), or mean ± SEM*.

* *p ≤ 0.05. BMI, body mass index; α-GI, α-glucosidase inhibitor; SGLT2, Sodium-glucose co-transporter-2 inhibitor; TC, total cholesterol; LDL-C, low-density lipoprotein cholesterol; HDL-C, high-density lipoprotein cholesterol; TG, triglycerides; RC, remnant cholesterol; FFA, free fatty acid*.

a *Chi-squared test or Fisher's exact test;*

b *Student's t-test;*

c *Mann-Whitney U-test*.

### Post-prandial Change in TRL and HDL Composition in Patients With CAD

**Figure 1:**
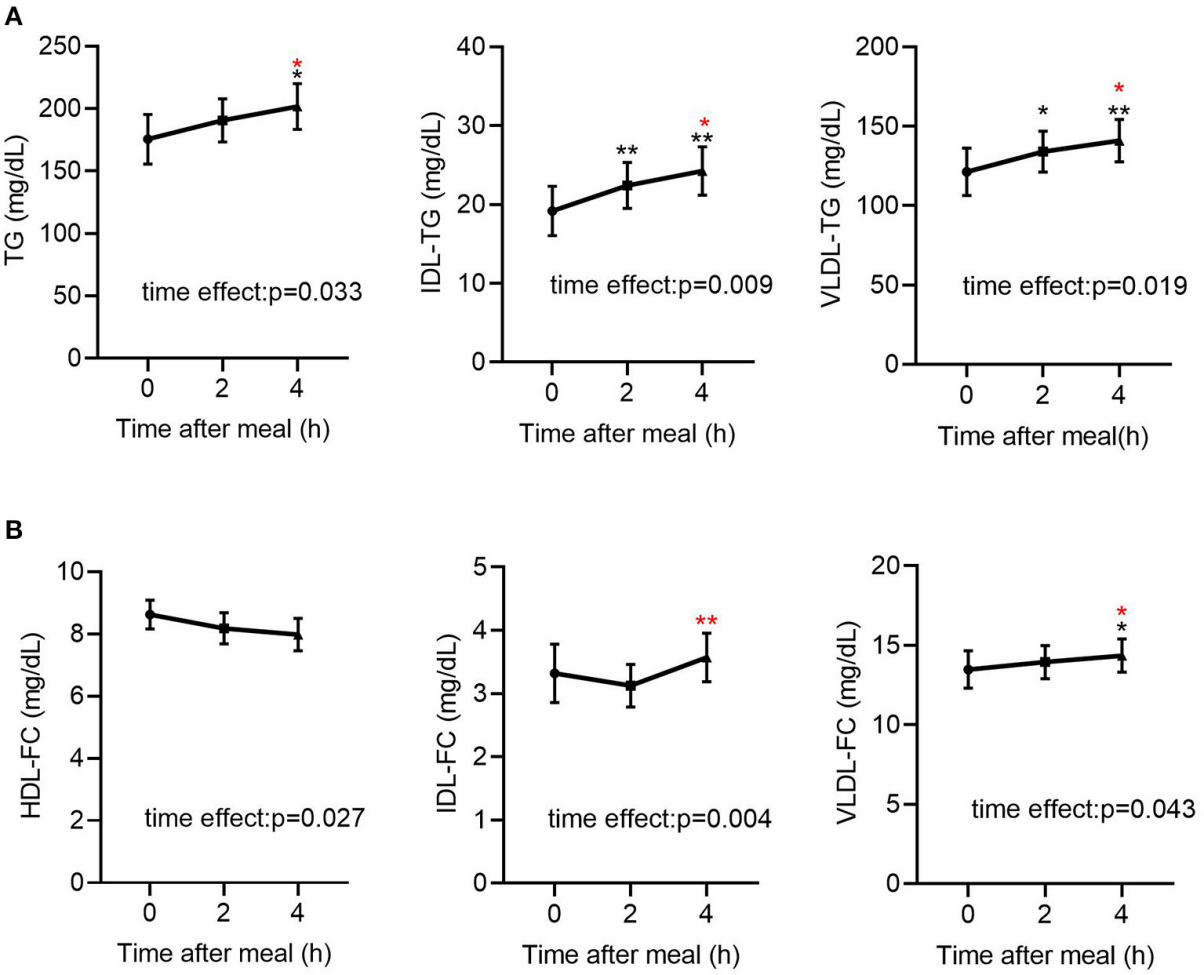
Fasting and post-prandial TG and FC levels in lipoproteins determined by NMR-based method in all subjects (*n* = 49). **(A)** Fasting and post-prandial TG, IDL-TG and VLDL-TG levels in all subjects (*n* = 49). **(B)** Fasting and post-prandial HDL-FC, IDL-FC, VLDL-FC levels in all subjects (*n* = 49). TG, triglyceride; FC, free cholesterol. Black indicates *p*-value when compared with fasting. Red indicates *p*-value when compared with 2 h post-prandial. *p ≤ 0.05; **p ≤ 0.01. Statistical significance was determined using Generalized Estimating Equations (TG, IDL-TG, VLDL-TG, IDL-FC, VLDL-FC) or repeated measurement ANOVA (HDL-FC). Bars represent mean ± SEM.

As shown in Table 2, in all participants, the post-prandial serum glucose and insulin levels were both elevated (*p* < 0.001 for both), peaked at 2 h after meals. Regarding the lipid profiles, the post-prandial serum TG level was significantly elevated [141.68 (80.94–199.37) vs. 161.82 (99.30–249.86) vs. 166.71 (102.10–289.56) mg/dL, *p* < 0.05] and the FFA levels were reduced [0.27 (0.19–0.43) vs. 0.10 (0.05–0.22) vs. 0.21 (0.07–0.39) mg/dL, *p* < 0.001], consistent with previous findings (20, 22). In addition, the TG levels in VLDL and IDL were significantly elevated (Table 2, Figure 1A). Post-prandial dyslipidemia, a potential residual risk factor, is a condition where TG-enriched TRLs are increased during the post-prandial period, especially cholesterol-enriched remnants. Thus, consistent with the increase in TG, total cholesterol in VLDL (VLDL-C) was significantly elevated [(28.93 ± 2.67) vs. (29.59 ± 2.36) vs. (31.23 ± 2.43) mg/dL, *p* < 0.01, Table 2]. Although the post-prandial difference in cholesterol in TRLs (TRL-C) was not statistically significant, the level of RC tended to increase, and it was considerably higher at 4 h than at 2 h [(21.10 ± 1.55) vs. (23.46 ± 1.69) mg/dL, *p* < 0.001].

**Table 2:** Fasting and post-prandial lipid profiles and metabolite concentrations in all the patients.

	**Time points after a meal**			
	**0 h**	**2 h**	**4 h**	***P*** **-value**
TC (mg/dL)	152.19 (129.53–180.09)	150.58 (127.01–177.11)	152.57 (130.74–176.04)	0.193
TG (mg/dL)	141.68 (80.94–199.37)	161.82 (99.30–249.86)	166.71 (102.10–289.56)	0.033^*^
HDL-C (mg/dL)	43.39 ± 1.13	43.55 ± 1.19	43.78 ± 1.11	0.741
IDL-C (mg/dL)	10.41 ± 1.37	9.32 ± 0.94	11.02 ± 1.11	<0.001^***^
LDL-C (mg/dL)	71.88 ± 4.59	72.62 ± 4.73	73.50 ± 4.49	0.408
VLDL-C (mg/dL)	28.93 ± 2.67	29.59 ± 2.36	31.23 ± 2.43	0.002^**^
TRL-C (mg/dL)	35.04 (20.85–50.53)	33.83 (22.19–51.89)	34.99 (24.20–47.59)	0.178
RC (mg/dL)	22.82 ± 2.09	21.10 ± 1.55	23.46 ± 1.69	<0.001^***^
Apo-A1 (mg/L)	121.02 ± 2.14	120.75 ± 2.22	120.91 ± 2.34	0.938
Apo-A2 (mg/L)	25.16 (22.80–27.02)	24.69 (22.63–27.26)	23.86 (23.04–28.47)	0.382
Apo-B (mg/L)	71.06 (59.63–86.09)	68.98 (57.07–85.03)	71.33 (60.69–89.62)	0.115
HDL-FC (mg/dL)	8.62 ± 0.46	8.18 ± 0.50	7.98 ± 0.52	0.027^*^
IDL-FC (mg/dL)	2.55 (1.49–4.22)	2.33 (1.46–4.56)	2.93 (1.50–4.96)	0.004^**^
IDL-TG (mg/dL)	12.99 (4.17–23.26)	17.59 (6.71–31.91)	18.73 (7.24–39.63)	0.009^**^
VLDL-FC (mg/dL)	12.14 (6.88–16.43)	12.42 (8.33–17.22)	13.21 (9.06–18.20)	0.043^*^
VLDL-TG (mg/dL)	102.37 (49.74–148.41)	118.33 (63.07–178.04)	116.27 (66.80–197.79)	0.019^*^
FFA (mmol/L)	0.27 (0.19–0.43)	0.10 (0.05–0.22)	0.21 (0.07–0.39)	<0.001^***^
Glucose (mmol/L)	4.60 (4.10–6.30)	7.30 (6.25–11.50)	6.00 (4.80–7.75)	<0.001^***^
Insulin (pmol/L)	134.98 (92.60–246.32)	815.71 (401.62–988.96)	398.36 (159.42–906.55)	<0.001^***^

*Data are shown as median (low quartile-upper quartile) or mean ± SEM*.

* *p ≤ 0.05;*

** *p < 0.01;*

*** *p < 0.001. Statistical significance was determined using Generalized Estimating Equations or repeated measurement ANOVA. TRL-C, triglyceride-rich lipoproteins cholesterol; VLDL-C, very-low-density lipoprotein cholesterol; VLDL-FC, very-low-density lipoprotein free cholesterol; IDL-C, intermediate-density lipoprotein cholesterol; IDL-FC, intermediate-density lipoprotein free cholesterol; HDL-FC, high-density lipoprotein free cholesterol; Apo-A1, apolipoprotein A-1; Apo-A2, apolipoprotein A2; Apo-B, apolipoprotein B*.

The total cholesterol in HDL (HDL-C) did not decrease significantly at the 2nd and 4th h (Table 2), in line with previous reports describing largely stable concentrations of HDL-C in the post-prandial phase (24, 25). Interestingly, the free cholesterol in HDL particles (HDL-FC) decreased significantly after a meal [(8.62 ± 0.46) vs. (8.18 ± 0.50) vs. (7.98 ± 0.52) mg/dL, *p* < 0.05], whereas in VLDL [12.14 (6.88–16.43) vs. 12.42 (8.33–17.22) vs. 13.21 (9.06–18.20) mg/dL, *p* < 0.05] and IDL [2.55 (1.49–4.22) vs. 2.33 (1.46–4.56) vs. 2.93 (1.50–4.96) mg/dL, *p* < 0.01], it increased markedly in the post-prandial phase (Figure 1B), suggesting the existence of a metabolic link between HDL-FC and TRLs.

**Figure 2:**
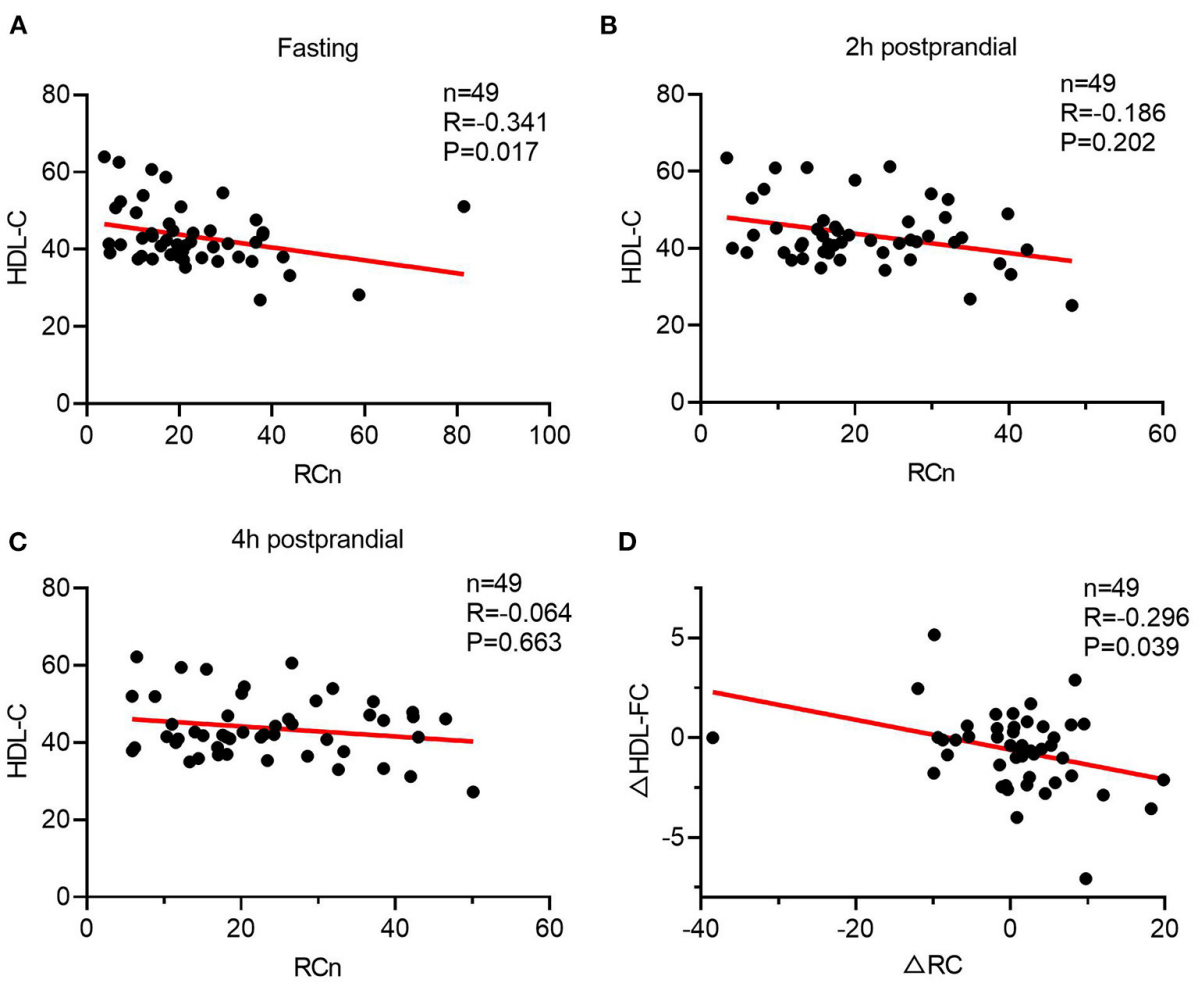
The Spearman's relationship between HDL lipids and the level of ANGPTL3. **(A–C)** The correlation of HDL-C and RC in a different time. **(A)** Fasting; **(B)** 2 h after a meal; **(C)** 4 h after a meal. **(D)** The Spearman's correlation of post-prandial changes in HDL-C and RC.

To further investigate the link between TRLs and HDL lipid content, the correlation between post-prandial cholesterol changes in TRLs and HDL was analyzed. First, fasting HDL-C and RC had a significant inverse correlation (Figure 2A); however, this correlation disappeared after a meal (Figures 2B,C). Subsequently, the results showed that decrease in the HDL-FC level was associated with increase in the RC level, further indicating the possible exchange of free cholesterol between HDL and TRLs after a meal (Figure 2D).

### Post-prandial Change in TRL and HDL Composition in CAD Patients With Abnormal TG Response to Diet

To investigate whether the link between HDL lipid content and TRLs depends on TRL lipolysis, this study analyzed the relationship between HDL lipids and the percentage of post-prandial plasma TG elevation (ΔTG), which partly represents the degree of TRL lipolysis. The post-prandial TRLs, as shown in Table 3, were all significantly elevated in patients with CAD with an abnormal TG response to diet (ΔTG > 30% group). In addition, RC increased markedly [(17.78 ± 2.22) vs. (19.28 ± 2.40) vs. (23.80 ± 2.93) mg/dL, *p* < 0.001, Table 3] after meals. Similarly, post-prandial RC in the ΔTG ≤ 30% group also increased significantly, although the increase was less significant than that in the ΔTG > 30% group (Table 4). Comparisons of post-prandial changes in HDL components were performed. In the ΔTG > 30% group, post-prandial components, such as FC, PL, and TGs, were dramatically altered in HDL. Notably, HDL-FC decreased considerably [(9.12 ± 0.78) vs. (8.12 ± 0.94) vs. (8.09 ± 1.06) mg/dL, *p* < 0.05] following a meal in the ΔTG > 30% group. In contrast, except for HDL-TG, the lipid content in HDL remained unchanged in the ΔTG ≤ 30% group (Table 4).

**Table 3:** Fasting and post-prandial lipid profiles and metabolite concentrations in ΔTG > 30% group.

	**Time points after a meal**			
	**0 h**	**2 h**	**4 h**	***P*** **-value**
TRL-C (mg/dL)	28.93 (16.90–47.68)	32.63 (18.40–42.12)	34.79 (25.43–45.55)	0.009^**^
HDL-C(mg/dL)	45.13 ± 2.09	46.52 ± 2.12	45.59 ± 1.94	0.261
IDL-C (mg/dL)	7.36 ± 1.06	8.70 ± 1.46	12.31 ± 2.03	0.007^**^
LDL-C (mg/dL)	74.26 ± 7.37	76.38 ± 7.50	74.99 ± 7.12	0.404
VLDL-C(mg/dL)	23.38 ± 3.21	27.75 ± 3.21	31.33 ± 3.57	<0.001^***^
RC (mg/dL)	17.78 ± 2.22	19.28 ± 2.40	23.80 ± 2.93	<0.001^***^
Apo-A1 (mg/L)	120.63 ± 3.62	120.54 ± 3.76	121.11 ± 4.07	0.820
Apo-A2 (mg/L)	24.83 (22.82–27.75)	25.07 (23.12–27.09)	25.86 (22.54–27.40)	0.665
HDL-FC (mg/dL)	9.12 ± 0.78	8.12 ± 0.94	8.09 ± 1.06	0.046^*^
HDL-PL (mg/dL)	61.71 ± 2.60	61.44 ± 2.51	63.12 ± 2.55	0.003^**^
HDL-TG (mg/dL)	10.72 ± 0.83	11.98 ± 0.85	13.87 ± 0.98	<0.001^***^
FFA (mmol/L)	0.21 (0.11–0.35)	0.08 (0.03–0.28)	0.19 (0.09–0.59)	0.001^**^
Glucose (mmol/L)	4.35 (4.10–5.18)	6.70 (5.78–7.78)	5.55 (4.75–6.55)	<0.001^***^
Insulin (pmol/L)	154.35 (94.15–248.85)	968.71 (535.04–992.89)	465.98 (172.93–986.97)	<0.001^***^

*Data are shown as median (low quartile-upper quartile) or mean ± SEM*.

* *p ≤ 0.05;*

** *p < 0.01;*

*** *p < 0.001. Statistical significance was determined using Generalized Estimating Equations or repeated measurement ANOVA. PL, phospholipid*.

**Table 4:** Fasting and post-prandial lipid profiles and metabolite concentrations in ΔTG ≤ 30% group.

	**Time points after a meal**			
	**0 h**	**2 h**	**4 h**	***P*** **-value**
TRL-C (mg/dL)	39.98 (27.63–60.87)	36.98 (24.86–53.86)	36.93 (22.53–48.77)	0.001^**^
HDL-C(mg/dL)	42.18 ± 1.24	41.50 ± 1.27	42.53 ± 1.30	0.172
IDL-C (mg/dL)	12.52 ± 2.13	9.76 ± 1.24	10.13 ± 1.25	0.027^*^
LDL-C (mg/dL)	70.23 ± 5.94	70.03 ± 6.17	72.48 ± 5.88	0.052
VLDL-C(mg/dL)	32.75 ± 3.82	30.86 ± 3.35	31.16 ± 3.34	0.260
RC (mg/dL)	26.29 ± 3.04	22.35 ± 2.04	23.21 ± 2.06	0.006^**^
Apo-A1 (mg/L)	121.29 ± 2.67	120.89 ± 2.78	120.76 ± 2.84	0.941
Apo-A2 (mg/L)	25.49 (22.75–26.75)	23.45 (22.45–27.33)	23.70 (23.11–28.74)	0.021^*^
HDL-FC (mg/dL)	8.28 ± 0.57	8.23 ± 0.56	7.90 ± 0.50	0.246
HDL-PL (mg/dL)	60.06 ± 1.75	59.41 ± 1.80	59.25 ± 1.77	0.504
HDL-TG (mg/dL)	12.97 ± 1.17	12.33 ± 1.13	11.69 ± 1.17	0.007^**^
FFA (mmol/L)	0.33 (0.24–0.47)	0.12 (0.05–0.20)	0.22 (0.04–0.37)	<0.001^***^
Glucose (mmol/L)	5.00 (4.15–6.75)	8.40 (6.45–13.50)	6.40 (4.85–8.65)	<0.001^***^
Insulin (pmol/L)	131.33 (91.59–249.28)	699.94 (349.05–980.88)	398.36 (151.25–631.16)	<0.001^***^

*Data are shown as median (low quartile-upper quartile), n (%), or mean ± SEM*.

* *p ≤ 0.05;*

** *p < 0.01;*

*** *p < 0.001. Statistical significance was determined using Generalized Estimating Equations or repeated measurement ANOVA*.

### Post-prandial Change in ANGPTL3 in CAD Patients With Abnormal TG Response to Diet

**Figure 3:**
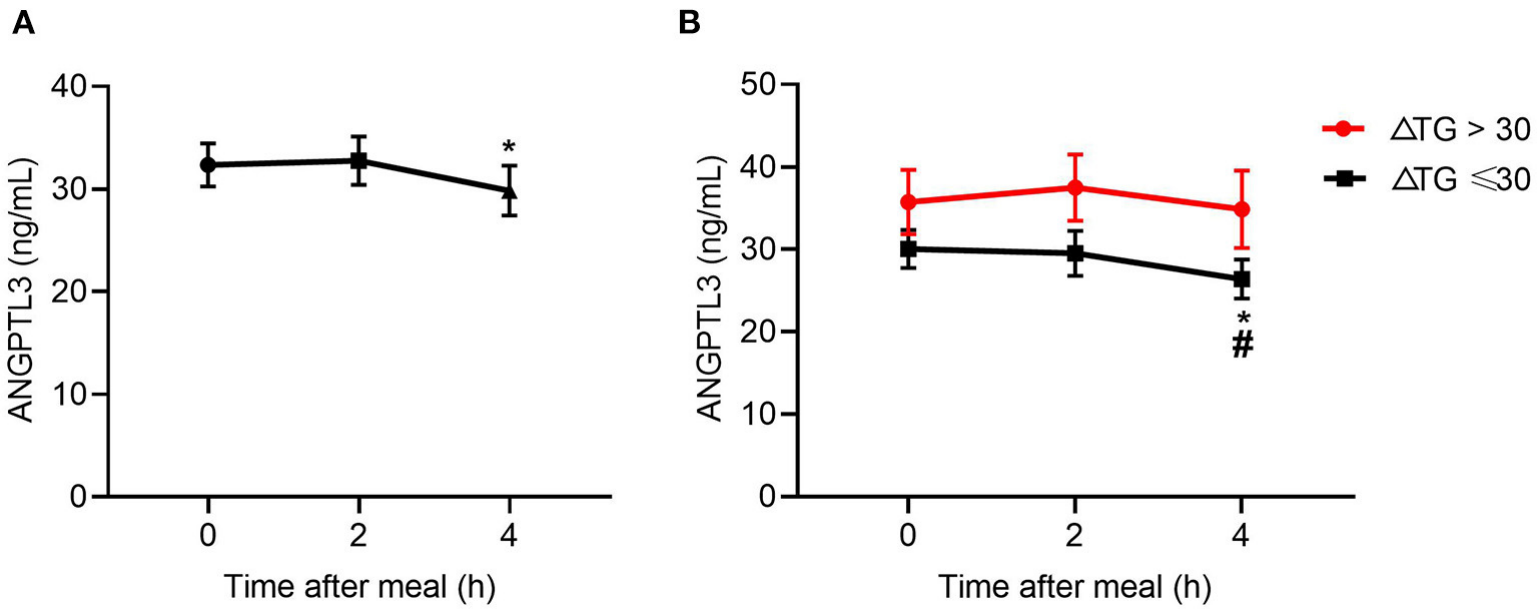
Changes in levels of ANGPTL3 at 2 and 4 h after a meal. **(A)** In all subjects (*n* = 49). **(B)** Changes in levels of ANGPTL3 at 2 and 4 h after a meal, according to ΔTG. *p ≤ 0.05 when compared with 2 h post-prandial, #*p* ≤ 0.05 when compared with fasting. Statistical significance was determined using Generalized Estimating Equations. Bars represent mean ± SEM.

The results above suggest a metabolic link between HDL-FC and TRLs, but the interplay between them is unclear. Considering the role of ANGPTL3 in HDL and TRL metabolism, this study investigated the change in ANGPTL3 in the study participants, showing that the ANGPTL3 levels tended to decrease after a meal (Figure 3A). Specifically, the ANGPTL3 levels in the ΔTG ≤ 30% group were markedly lower than those in the ΔTG > 30% group (*p* < 0.05), and only in the ΔTG ≤ 30% group, the 4-h post-meal ANGPTL3 level was markedly lowered [26.78 (19.10–35.95) vs. 20.84 (17.06–35.04) ng/mL, *p* < 0.05; Figure 3B]. Decreased post-prandial ANGPTL3 levels mitigate the inhibition of LPL activity and promote post-prandial TG lipolysis, which may provide feedback regulation of feeding in a physiological state.

The relationship between HDL lipids and the level of ANGPTL3, a lipolysis inhibitor, is worth exploring. As shown in Table 5, both the HDL-C (*r* = 0.369, *p* < 0.01 fasting; *r* = 0.324, *p* < 0.05, post-prandial 2 h, *r* = 0.380, *p* < 0.01, post-prandial 4 h) and the HDL-FC (*r* = 0.257, *P* = 0.074, fasting; *r* = 0.428, *P* < 0.01, post-prandial 2 h, *r* = 0.471, *p* < 0.01, post-prandial 4 h) levels were positively correlated with post-prandial serum ANGPTL3, suggesting that post-prandial ANGPTL3 is related to the homeostasis of HDL metabolism. Interestingly, in contrast to HDL-C, the correlation between post-prandial HDL-FC and ANGPTL3 was stable regardless of the change in post-prandial TG.

**Table 5:** The Spearman's correlation between ANGPTL3 and HDL-C, HDL-FC levels.

	**Fasting spearman R spearman R (** ***P*** **-value)**			**2 h post-prandial spearman R (** ***P*** **-value)**			**4 h post-prandial spearman R (** ***P*** **-value)**		
	**All**	**ΔTG ≤ 30%**	**ΔTG > 30%**	**All**	**ΔTG ≤ 30%**	**ΔTG > 30%**	**All**	**ΔTG ≤ 30%**	**ΔTG > 30%**
HDL-C	0.369^**^ (0.009)	0.216 (0.261)	0.555^*^ (0.011)	0.324^*^ (0.023)	0.068 (0.726)	0.611^**^ (0.004)	0.380^**^ (0.007)	0.255 (0.182)	0.514^*^ (0.020)
HDL-FC	0.257 (0.074)	0.144 (0.457)	0.450^*^ (0.047)	0.428^**^ (0.002)	0.412^*^ (0.027)	0.508^*^ (0.022)	0.471^**^ (0.001)	0.497^**^ (0.006)	0.505^*^ (0.023)

* *p ≤ 0.05;*

** *p < 0.01. All statistics means incorporating data of fasting, 2 h post-prandial and 4 h post-prandial HDL-C, HDL-FC, and ANGPTL3 into statistical analysis*.

As shown in Supplementary Figure 1, compared with the ΔTG > 30% group, there was a trend for an increase in glucose levels in the ΔTG ≤ 30% group [fasting: 5.00 (4.15–6.75) vs. 4.35 (4.10–5.18) mmol/L, *p* = 0.089; post-prandial 2 h: 8.40 (6.45–13.50) vs. 6.70 (5.78–7.78), *p* = 0.058]. This trend is opposite to ANGPTL3 levels, suggesting that there may be a negative correlation between blood glucose and ANGPTL3 levels. Thus, to further investigate the factors underlying the feedback mechanism of ANGPTL3 regulation, this study analyzed the association between ANGPTL3 and glucose, insulin levels as well as lipid profiles in all participants (Table 6). Interestingly, in the fasting state, the glucose levels correlated with the levels of ANGPTL3 (*r* = −0.358, *p* < 0.05). Moreover, both the fasting and post-meal ANGPTL3 levels were negatively correlated with post-meal changes in glucose levels (*r* = −0.304 for fasting, *p* < 0.05; *r* = −0.344 for post-meal, *p* < 0.05). However, there was no significant correlation between insulin and ANGPTL3 levels.

**Table 6:** The Spearman's correlation between ANGPTL3 and lipid profiles, glucose, and insulin levels.

	**Fasting spearman R (** ***P-*** **value)**			**2 h post-prandial spearman R (** ***P*** **-value)**			**4-h post-prandial spearman R (** ***P*** **-value)**		
	**All**	**ΔTG ≤30%**	**ΔTG > 30%**	**All**	**ΔTG ≤30%**	**ΔTG > 30%**	**All**	**ΔTG ≤30%**	**ΔTG > 30%**
TG	−0.187 (0.198)	0.150 (0.437)	−0.487^*^ (0.029)	−0.083 (0.571)	0.172 (0.371)	−0.547^*^ (0.012)	−0.107 (0.464)	−0.007 (0.972)	−0.284 (0.225)
TC	0.271 (0.060)	0.067 (0.732)	0.513^*^ (0.021)	0.396^**^ (0.005)	0.337 (0.074)	0.501^*^ (0.025)	0.452^**^ (0.001)	0.423^*^ (0.022)	0.469^*^ (0.037)
LDL-C	0.238 (0.099)	−0.026 (0.893)	0.486^*^ (0.030)	0.251 (0.082)	0.047 (0.808)	0.441 (0.052)	0.348^*^ (0.014)	0.225 (0.240)	0.389 (0.090)
VLDL-C	−0.209 (0.149)	0.072 (0.709)	−0.424 (0.062)	−0.071 (0.630)	0.172 (0.371)	−0.408 (0.075)	−0.107 (0.465)	−0.045 (0.817)	−0.179 (0.450)
IDL-C	−0.058 (0.690)	0.142 (0.461)	−0.168 (0.478)	−0.031 (0.834)	0.119 (0.538)	−0.206 (0.384)	0.029 (0.845)	0.087 (0.653)	−0.042 (0.860)
RC	−0.183 (0.208)	0.041 (0.831)	−0.340 (0.143)	−0.041 (0.777)	0.133 (0.492)	−0.224 (0.342)	−0.013 (0.928)	0.029 (0.881)	−0.059 (0.806)
LDL-FC	0.212 (0.145)	−0.126 (0.514)	0.565^**^ (0.009)	0.294^*^ (0.040)	0.127 (0.513)	0.531^*^ (0.016)	0.338^*^ (0.017)	0.278 (0.144)	0.438 (0.054)
VLDL-FC	−0.200 (0.168)	0.097 (0.617)	−0.400 (0.081)	−0.072 (0.624)	0.172 (0.373)	−0.437 (0.054)	−0.098 (0.504)	−0.020 (0.917)	−0.167 (0.482)
IDL-FC	−0.097 (0.508)	0.126 (0.514)	−0.250 (0.289)	−0.048 (0.745)	0.152 (0.430)	−0.263 (0.264)	−0.007 (0.962)	0.047 (0.810)	−0.075 (0.753)
Glucose	−0.358^*^ (0.012)	−0.350 (0.063)	−0.302 (0.196)	−0.294^*^ (0.040)	−0.097 (0.618)	−0.539^*^ (0.014)	−0.261 (0.071)	−0.098 (0.615)	−0.524^*^ (0.018)
Insulin	−0.254 (0.081)	−0.138 (0.476)	−0.430 (0.066)	−0.126 (0.393)	−0.164 (0.397)	−0.321 (0.180)	−0.260 (0.071)	−0.238 (0.213)	−0.385 (0.094)

* *p ≤ 0.05;*

** *p < 0.01*.

MLR analysis was utilized to explore possible physiological factors that may influence the ANGPTL3 levels. The equation incorporated ANGPTL3, fasting lipid profiles, glucose levels, and FFA levels. We found that fasting glucose is an independent contributor to the ANGPTL3 levels (Supplementary Table 2).

### Post-prandial Change in Glucose May Affect Circulating ANGPTL3

**Figure 4:**
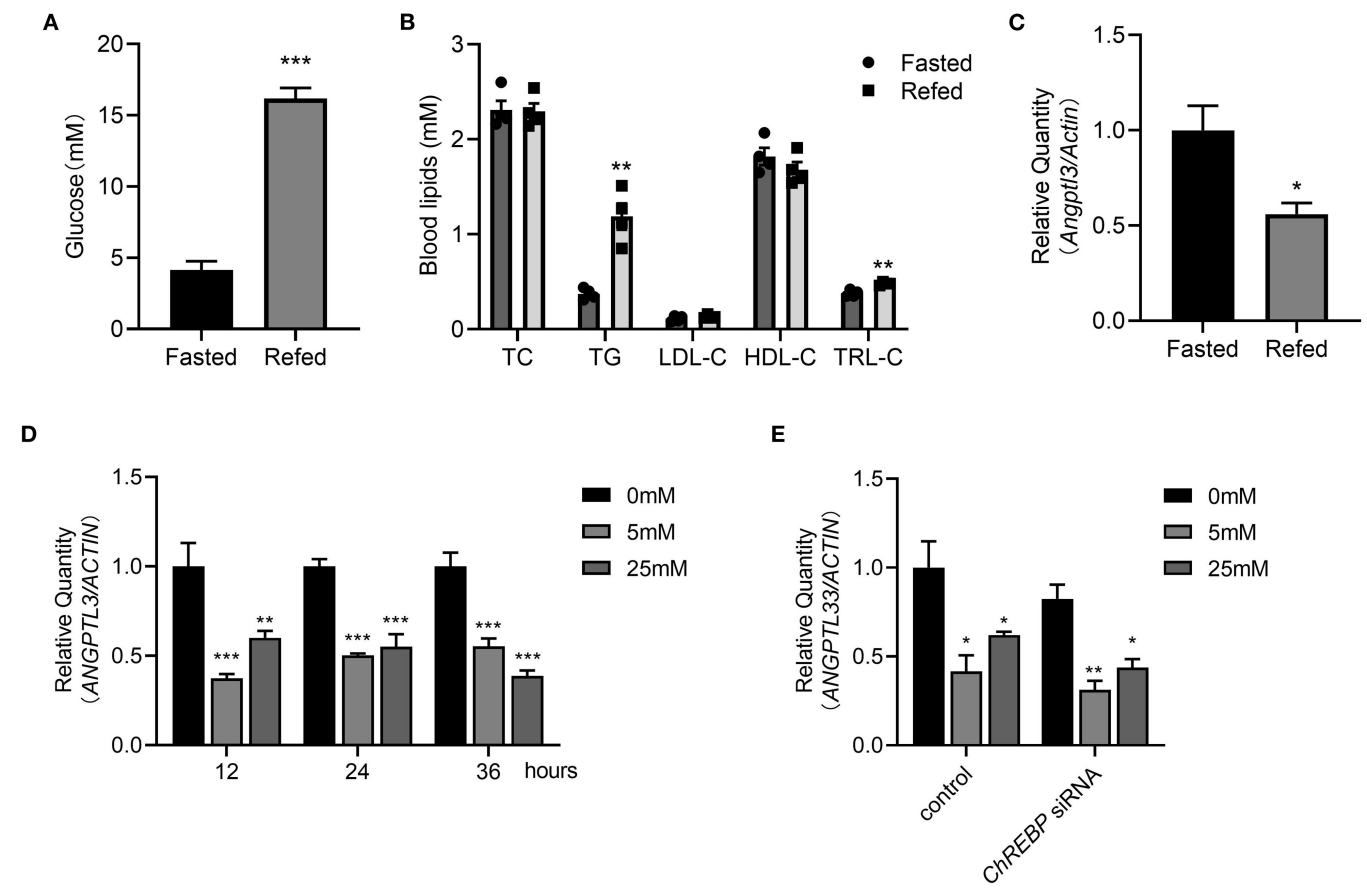
Post-prandial change of glucose may affect the ANGPTL3 expression. **(A)** Eight-week-old male WT mice were fasted for 16 h, or fasted for 16 h and then refed a high-carbohydrate, low-fat diet for 3 h. The blood glucose levels in mice were measured (*n* = 4 per group). **(B)** The blood lipids profiles in mice (*n* = 4 per group) treated as in **(A)**. **(C)** qPCR analysis of the *Angptl3* mRNA in mice livers (*n* = 4 per group) treated as in **(A)**. **(D)** Relative expression of *ANGPTL3* in HepG2 cells, which were fasted for 12h, and then treated with 0, 5, and 25 mM glucose for 12 h (*n* = 3 per group). **(E)** Relative expression of *ANGPTL3* in HepG2 cells transfected with ChREBP siRNA or control in different glucose concentrations (*n* = 3 per group). *p ≤ 0.05; **p < 0.01; ***p < 0.001; Statistical significance was determined using unpaired two-tailed Student's *t*-test **(A–C)** and two-way ANOVA with multiple comparisons **(D,E)**. Bars represent mean ± SEM.

As previously stated, the associations cannot necessarily implicate causal linkage. *In vivo* and *in vitro* experiments were conducted to obtain deeper insight into the potential causality between glucose and ANGPTL3 expression. First, after 16 h of fasting and 3 h of refeeding both the post-prandial glucose and TG levels were considerably greater than those after fasting (Figures 4A,B). Simultaneously, hepatic expression of *Angptl3* was drastically reduced (Figure 4C). To demonstrate that ANGPTL3 expression is sensitive to stimulation by glucose concentration and to exclude the possibility that the effect of liver ANGPTL3 expression in the *in vivo* refeeding experiment occurred because of the change in post-prandial insulin level, as previously reported (26), HepG2 cells were cultured in insulin-free DMEM with no glucose and then with high glucose (25 mM) or low glucose (5 mM) for various time points. The expression of *ANGPTL3* was markedly inhibited after adding glucose (Figure 4D), indicating that post-prandial glucose is an underlying inhibitor of ANGPTL3. The carbohydrate response element-binding protein (ChREBP) is a glucose-activated transcription factor that links glucose and lipid metabolism (27, 28). As shown in Figure 4E, after effective knockdown of ChREBP, the expression of *ANGPTL3* was inhibited by glucose supplementation, implying the existence of other unknown mechanisms responsible for ANGPTL3 regulation.

## Discussion

This study demonstrated changes in post-prandial HDL and TRL components in patients with CAD receiving statin treatment. Consistent with the findings of earlier literature, there remained a peak in the TG concentration after a meal. Despite the negative relationship between TG and HDL-C levels shown by multiple studies (29, 30), the NMR-based method assessment of post-prandial total cholesterol in HDL particles (HDL-C) did not show this decrease in our study. Although HDL-C was not significantly altered, the free cholesterol in HDL particles (HDL-FC) was markedly decreased after a meal, consistent with previous findings (31). Moreover, the change in HDL-FC was negatively associated with the shift in RC levels, indicating a link between free cholesterol in HDL and TRLs. Nevertheless, the mechanisms of the metabolic association between HDL and TRLs remain uncertain. Previous studies have suggested that an increase in TG concentration lowers HDL-C levels by enhancing HDL-TG clearance under EL lipolysis (32, 33). In the hypertriglyceridemic state, the CETP-mediated exchange of TGs and cholesteryl esters between HDL and TRLs is also important for maintaining the HDL-C levels (34, 35). Some studies have revealed that HDL facilitates TRL removal by accelerating lipolytic-free cholesterol transfer from TRL to HDL, which could reduce cholesterol influx into the arterial wall (9). Indeed, this study suggests that in patients with CAD in whom the 4-h post-meal HDL-FC was decreased, the post-prandial TG and RC level were increased significantly, indicating that HDL-FC might be linked to RC metabolism. Feng et al. (36) found that a decrease in HDL-FC may represent impaired TRLs lipolysis. TRL generation and lipolysis increased after a meal. During TRLs lipolysis, high amounts of free cholesterol in TRLs are delivered to HDL, leading to marked elevation in HDL-FC (35). Hence, post-prandial decreases of HDL-FC in patients with CAD might indicate impaired TRL lipolysis. Thus, whether HDL-FC can be used as a marker of impaired post-prandial lipolysis is worth investigating.

Regarding post-prandial lipolysis, some researchers have found that ANGPTL3 is a key inhibitor of LPL activity (12). In this study, the levels of ANGPTL3 at the fasting and post-prandial phases were investigated in patients with CAD who were using statins. Interestingly, the baseline and post-prandial ANGPTL3 levels were significantly lower in the normal TG response group than in the exaggerated TG response group. A higher ANGPTL3 level corresponds to lower LPL activity, resulting in a higher post-prandial TG level. The difference in ANGPTL3 level may partly explain the pattern of the TG response after a meal.

Previous studies have found a link between ANGPTL3 and HDL-C levels (15, 17, 37). Mechanistically, ANGPTL3 reduces plasma HDL-C levels by inhibiting phospholipase EL, which hydrolyzes HDL-PL (16). However, for the first time, the post-prandial decrease in ANGPTL3 was shown to be significantly correlated with post-meal HDL-FC levels, suggesting that ANGPTL3 may act as an intermediary between TRL lipolysis and HDL-FC. It could be speculated that under physiological conditions, ANGPTL3 inhibits LPL-mediated lipolysis and reduces the transfer of free cholesterol from TRLs to HDL, thereby reducing the HDL-FC levels. However, the possibility that ANGPTL3 affects the HDL-C levels by promoting reverse cholesterol transport (RCT) or RRT cannot be excluded. Indeed, a very recent animal study using an *Angptl3* antisense oligonucleotide showed that inhibition of ANGPTL3 improved HDL-mediated RCT *in vivo* (38). Further *in vivo* RCT trials in *Angptl3*^−/−^ mice are required.

The mechanism of post-prandial decrease of ANGPTL3 needs to be ascertained. There is evidence that the ANGPTL3 levels are regulated in the short term following metabolic stress. Zhao et al. (17) revealed that the ANGPTL3 levels in female patients with type-2 diabetes mellitus were considerably lower than those in female patients without diabetes, indicating that glucose metabolism may affect the ANGPTL3 levels. Interestingly, this study found that the fasting glucose levels were associated with post-meal decrease in ANGPTL3 levels. Moreover, *in vivo* and *in vitro* studies have shown that glucose could downregulate the expression of hepatic ANGPTL3 independently of ChREBP, which may partially explain the post-meal change in ANGPTL3 levels. However, a recent study showed that glucose and dietary fatty acids had no effect on ANGPTL3, while omega-3/-6-polyunsaturated fatty acids could inhibit ANGPTL3 expression in adipocytes (39), implying the tissue-specific regulation of ANGPTL3. Furthermore, the disappearing decrease in ANGPTL3 levels in the ΔTG > 30% group observed in this study suggests that the regulation of ANGPTL3 is a complex process. In addition to glucose, other substrates may be involved in ANGPTL3 regulation. Insulin and leptin (26, 40) also participate in the regulation of ANGPTL3. However, we did not find the correlation between insulin and ANGPTL3 levels in our study. Therefore, further mechanism is worth exploring.

This study has several strengths. The main strength comprises the first demonstration that post-prandial ANGPTL3 could be regulated by changes in blood glucose levels and might interplay with the cholesterol transfer of TRLs and HDL after a meal. Furthermore, this study provided a complete assessment based on participants' daily life conditions, resulting in more generalizable results. However, this study had several limitations. It is worth noting that ANGPTL3 and ANGPTL8 always form a complex in circulation, and the LPL-inhibitory activity of ANGPTL3/8 is >100-fold more potent than that of ANGPTL3 alone (41). Thus, the function of ANGPTL3 cannot be fully related to its level. Additionally, TG-AUC (TG area under curve) might be a more precise method to characterize post-prandial TG response than post-prandial TG peak values or incremental percentage used in our study (42, 43). However, it is difficult for us to determine precise TG-AUC due to limited time points of post-prandial blood lipid determination. Finally, this study was exploratory, and as a result of the design constraints, it was correlative, not permitting causal inference. Therefore, additional studies with larger cohorts are required to corroborate these results.

In summary, in patients with CAD using statins, ANGPTL3 may be an important regulator of post-prandial TG and HDL components. Therapy targeting ANGPTL3 could prove promising for both TG and HDL metabolism.

## Data Availability Statement

The original contributions presented in the study are included in the article/Supplementary Materials, further inquiries can be directed to the corresponding authors.

## Ethics Statement

The studies involving human participants were reviewed and approved by the Medical Ethics Committee of the Second Xiangya Hospital of Central South University. The patients/participants provided their written informed consent to participate in this study. The animal study was reviewed and approved by the Institutional Animal Care and Use Committees of the Central South University.

## Author Contributions

BY conceptualized, designed, and interpreted the study. XG, ZH, and JC conducted experiments. ZH and JC generated figures and tables. BY, DH, JH, and ZH analyzed the data and wrote the article. DH, ZH, and JC provided the technical support. DP contributed to the discussion of the project and paper. All authors contributed to the article and approved the submitted version.

## Funding

This work was supported in whole or in part by the National Natural Science Foundation of China (Grant Nos. 82170483 to BY and 82100496 to DH) and Chinese Cardiovascular Association-Access fund (Grant Nos. 2020-CCA-ACCESS-067 to DH and 2020-CCA-ACCESS-070 to BY).

## Conflict of Interest

The authors declare that the research was conducted in the absence of any commercial or financial relationships that could be construed as a potential conflict of interest.

## Publisher's Note

All claims expressed in this article are solely those of the authors and do not necessarily represent those of their affiliated organizations, or those of the publisher, the editors and the reviewers. Any product that may be evaluated in this article, or claim that may be made by its manufacturer, is not guaranteed or endorsed by the publisher.
