# Supplementary material for: ANGPTL3 Is Involved in the Post-prandial Response in Triglyceride-Rich Lipoproteins and HDL Components in Patients With Coronary Artery Disease
Source: Front Cardiovasc Med. 2022 Jun 29;9:913363. doi: 10.3389/fcvm.2022.913363 (PMC9276986; doi:10.3389/fcvm.2022.913363)
Supplement: Supplementary file 1 [file Data_Sheet_1.doc]

**Supplementary Table 1 Sequences of PCR primer pair used in this study**

| Gene | Species | Primer sequences (5' to 3') |
| --- | --- | --- |
| *β-ACTIN* | Human | Forward: CATGTACGTTGCTATCCAGGC  Reverse: CTCCTTAATGTCACGCACGAT |
| *ANGPTL3* | Human | Forward: ATTTTAGCCAATGGCCTCCTTC  Reverse: CTGGTTTGCAGCGATAGATCATA |
| *ChREBP* | Human | Forward: CCAGCCTCAAGGTGAGCAAA  Reverse: CACGCTCCTGCTGTAGCA |
| *Actin* | Mice | Forward: GAGACCTTCAACACCCCAGC  Reverse: ATGTCACGCACGATTTCCC |
| *Angptl3* | Mice | Forward: TCTACTGTGATACCCAATCAGGC  Reverse: CATGTTTCGTTGAAGTCCTGTGA |

**Supplementary Table 2** The independent contribution of lipids and glucose to fasting ANGPTL3 levels.

| **Liner-regression covariates** | **All** | | **△TG≤30% group** | | **△TG>30% group** | |
| --- | --- | --- | --- | --- | --- | --- |
|  | **Standard β** | ***P*-value** | **Standard β** | ***P*-value** | **Standard β** | ***P*-value** |
| Glucose | -0.402^**^ | 0.008 | -0.419 | 0.073 | -0.547^**^ | 0.004 |
| TG | -0.138 | 0.326 | 0.116 | 0.557 | -0.628^**^ | 0.003 |
| TC | 0.357^**^ | 0.009 | 0.222 | 0.317 | 0.394^*^ | 0.035 |
| FFA | -0.088 | 0.565 | -0.090 | 0.714 | 0.264 | 0.202 |

Serum ANGPTL3, TC, TG, glucose and FFA were log-transformed to be normally distributed. **p* ≤ 0.05; ***p* < 0.01; ****p* < 0.001.


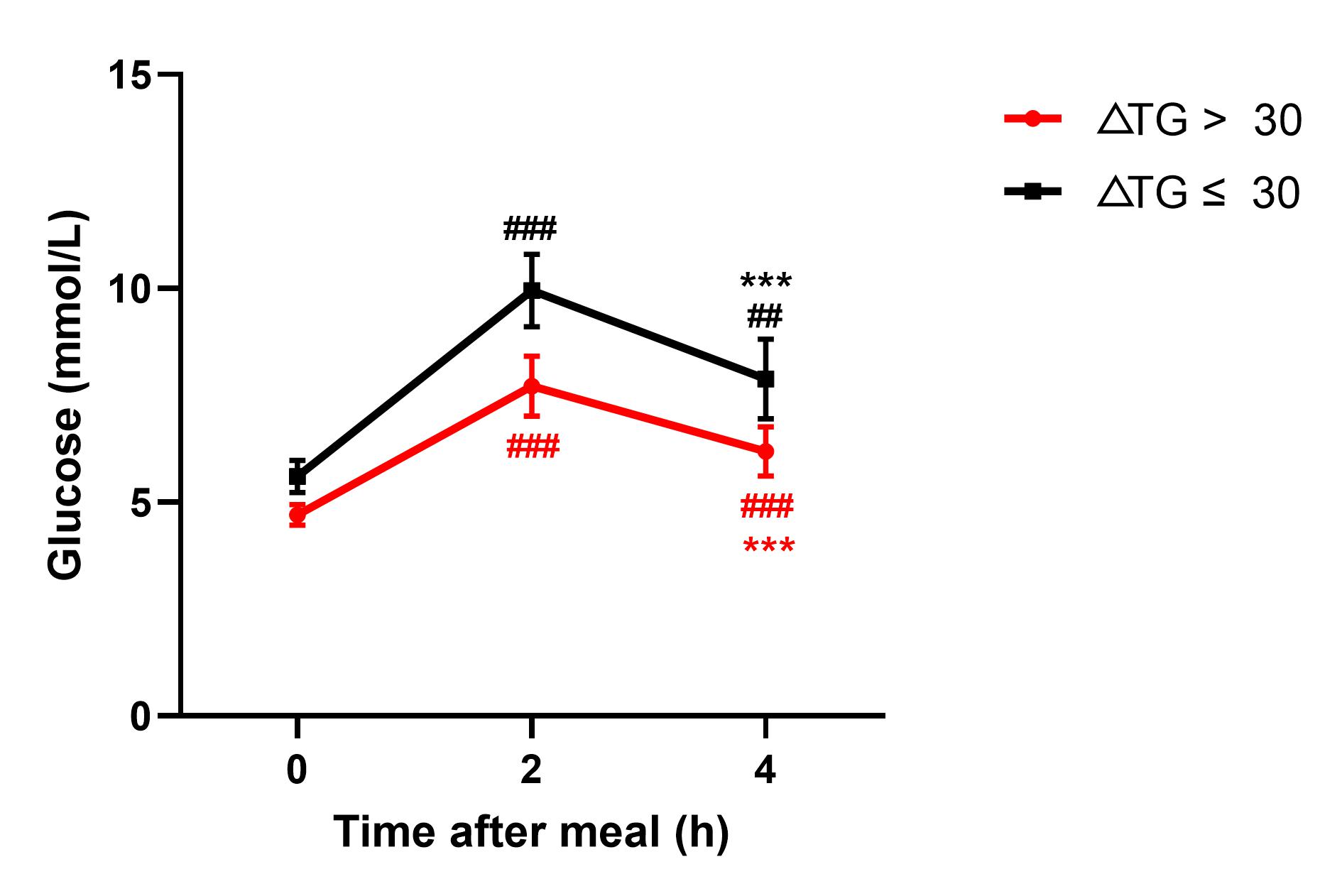


**Supplementary Figure 1.** Changes in levels of glucose at 2 h and 4 h after a meal according to △TG. **p* ≤ 0.05, ***p* < 0.01, ****p* < 0.001, when compared with 2 h postprandial; #*p* ≤ 0.05, ##*p* < 0.0, and ###*p* < 0.001, when compared with fasting. Statistical significance was determined using generalized estimating equations. Bars represent mean ± SEM.
